# Electro-magnetic field promotes osteogenic differentiation of BM-hMSCs through a selective action on Ca^2+^-related mechanisms

**DOI:** 10.1038/srep13856

**Published:** 2015-09-14

**Authors:** Loredana Petecchia, Francesca Sbrana, Roberto Utzeri, Marco Vercellino, Cesare Usai, Livia Visai, Massimo Vassalli, Paola Gavazzo

**Affiliations:** 1 Institute of Biophysics, National Research Council, Via De Marini 6, 16149 Genova, Italy; 2 Institute for Macromolecular Studies, National Research Council, Via De Marini 6, 16149 Genova, Italy; 3 Dept. of Molecular Medicine, Centre for Health Technologies (C.H.T.), INSTM UdR of Pavia, University of Pavia, Italy; 4 Dept. of Occupational Medicine, Ergonomy and Disability, Laboratory of Nanotechnology, Salvatore Maugeri Foundation, IRCCS, Pavia, Italy

## Abstract

Exposure to Pulsed Electromagnetic Field (PEMF) has been shown to affect proliferation and differentiation of human mesenchymal stem cells derived from bone marrow stroma (BM-hMSC). These cells offer considerable promise in the field of regenerative medicine, but their clinical application is hampered by major limitations such as poor availability and the time required to differentiate up to a stage suitable for implantation. For this reason, several research efforts are focusing on identifying strategies to speed up the differentiation process. In this work we investigated the *in vitro* effect of PEMF on Ca^2+^-related mechanisms promoting the osteogenic differentiation of BM-hMSC. Cells were daily exposed to PEMF while subjected to osteogenic differentiation and various Ca^2+^-related mechanisms were monitored using multiple approaches for identifying functional and structural modifications related to this process. The results indicate that PEMF exposure promotes chemically induced osteogenesis by mechanisms that mainly interfere with some of the calcium-related osteogenic pathways, such as permeation and regulation of cytosolic concentration, leaving others, such as extracellular deposition, unaffected. The PEMF effect is primarily associated to early enhancement of intracellular calcium concentration, which is proposed here as a reliable hallmark of the osteogenic developmental stage.

Stem cells are present in all multicellular organisms and have acquired an important role in regenerative medicine because of their capacity to self renew or differentiate towards several specific cell lineages. These multipotent cells have been attracting considerable research interest for a number of years now^1^. Numerous studies have provided evidence of the tissue reparatory properties of BM-MSCs and have improved their stability in culture, promoting them as suitable candidates for many therapeutic applications.

BM-hMSCs are able to differentiate towards condrogenic, osteoblastic or adipose mesenchymal lineages. Differentiation towards the osteoblastic lineage has aroused great interest because, *in vitro,* it resembles a multistep physiological process. According to a scheme proposed in a rodent model of calvaria osteoblasts, bone maturation comprises three successive phases spanning a period of about 30 days: proliferation, matrix maturation and matrix mineralization^2^. Similarly, three developmental phases have been recognized in BM-hMSC osteogenesis by means of genomic profiling analysis^3^,^4^.

Exposure to Pulsed Electromagnetic fields (PEMF) has been shown to affect cell proliferation and differentiation by influencing multiple metabolic pathways depending on lineage and maturation stage. In the osteoblast lineage, PEMF contributes to bone formation induced by demineralized bone matrix and stimulates fracture healing^5^, probably through the action of progenitors that are already committed towards bone. In calvarial cells, PEMF seems to contribute to proliferation but not differentiation^6^, while there are indications that it modulates the behavior of MSCs by promoting differentiation towards the osteoblastic lineage initially triggered by BMP2^5^ or by the roughness of the growing surface^7^.

Exposure of biomolecules such as DNA and proteins to electromagnetic fields can produce conformational changes arising from alterations in charge distribution. These changes have a crucial impact on membrane transport proteins, including ion channels, probably accounting for the biological effects induced in cells by the application of weak PEMFs^8^. To obtain a biological response, the electromagnetic signal has to be transduced into a biological signal and there is general agreement on considering intracellular Ca^2+^ as one of the main actors of cell fate specification^9^.

The concentration of intracellular free calcium capable of triggering cellular signaling pathways is related to calcium ions entering specialized transporters in the plasma membrane and their release from internal stores, primarily endoplasmic reticulum (ER). Ca^2+^ entry across the membrane is mediated by various actors, including voltage-gated Ca channels (VGCC), expressed in excitable cells such as neurons and myocytes as well as in non-excitable cell lines, including rat osteoblasts and chondrocytes^10^. There is growing evidence that a pivotal role in the movement of Ca^2+^ in osteoblasts is played by VGCCs of the L-type, which couple membrane depolarization to calcium entry and are modulated by hormones, vitamin D and mechanical stimulation^11^,^12^. Only a small population of BM-hMSC expresses L-type VGCC^13^,^14^,^15^ and, according to some authors, the effect of PEMF is preferentially mediated by L-type VGCC in many cell types, including stem cells^16^.

The work reported here sought to characterize the effect of daily exposure of BM-hMSC to a pulsed electromagnetic field (PEMF) during *in vitro* osteogenesis, with special focus on alterations of Ca^2+^-related aspects of cell metabolism. A range of biophysical approaches were adopted to provide a comprehensive description of the modifications triggered by PEMF.

## Materials and Methods

### Cell cultures

BM-hMSCs were isolated as previously described^17^. For this study we have used mainly cells at passage 3. For proliferation, cells were cultured at 37 °C in a humidified incubator with 5% CO_2_ in maintenance medium (MM), low-glucose DMEM (Dulbecco’s modified Eagle’s) supplemented with 10% FBS, 1% glutamine, 50 μg/ml penicillin-streptomycin and amphotericin B (Lonza Group Ltd.). To induce osteogenesis, BM-hMSCs were maintained in Osteogenic Differentiating Medium (ODM), α-MEM (Minimum Essential Medium) supplemented with 10% FBS, antibiotics and the osteogenic mixture containing 100nM dexamethasone, 5 mM β-glycerophosphate disodium and 50 mg/ml ascorbic acid (Sigma-Aldrich, S. Louis, MO, USA). Treatment lasted up to 27 days and the medium was changed every 3 days. The study was conducted in accordance with the Review Board of Fondazione IRCCS Policlinico San Matteo and the University of Pavia (2011).

### Pulsed electromagnetic field (PEMF)

The electromagnetic apparatus consisted of a supporting structure custom-designed in a tube of polymethylmethacrylate; the windowed tube carried a well-plate and two solenoids, the Helmoltz coils, the planes of which were parallel^17^.

The stimulation protocol was selected on the basis of previous results^18^: cells were exposed to PEMF for 10 min each day at the same time and the following parameters were adopted: magnetic field 2 ± 0.2 mT, induced electronic tension amplitude 5 ± 1 mV, frequency of 75 ± 2 Hz, pulse duration 1.3 ms.

### MTT test

To evaluate the mitochondrial activity of cultured cells, a test with 3-(4,5-dimethylthiazole-2-yl)-2,5-diphenyl tetrazolium bromide (MTT; Sigma-Aldrich, St. Louis, MO) was performed on days 3, 9, 15, 21, 27 on control cells or cells exposed to different stimuli: electromagnetic field alone (ODM−/PEMF+), differentiating medium alone (ODM+/PEMF–) or both stimuli (ODM+/PEMF+).

### Apoptosis

The Annexin V-FITC Apoptosis Detection Kit (Bender Medsystems, Vienna, Austria) was used according to the manufacturer’s instructions, on the same samples as above.

### Osteogenic staining

Mineral deposition was assessed by staining with Alizarin Red S^19^. Cell populations were fixed in 4% paraformaldehyde (PFA), and stained with 2% aqueous solution of Alizarin red S (Sigma Aldrich) for 10 minutes at room temperature. Then cells were examined under an upright microscope.

### Morphological measurements

Brightfield microscopy was conducted using an upright Nikon Ni-U light microscope equipped with a Nikon DS-Fi2 color CCD camera and operated at 40x magnification. All images were pre-processed with the open source software ImageJ [http://imagej.nih.gov/ij/index.html] and a fully automated analysis of cell orientation was obtained by means of the plugin OrientationJ^20^. This procedure calculates the local orientation of identified morphological features and provides a color-coded map of this parameter, along with the full distribution of orientations.

### Atomic force microscopy imaging

Atomic force microscopy (AFM) imaging was performed using a Nanowizard II system (JPK Instruments Gmbh, Berlin, Germany) mounted on top of a Zeiss Axiovert inverted optical microscope and equipped with a 100 μm lateral range and 15 μm vertical range scanner. AFM imaging was performed in non-contact mode using NCHR cantilevers (NanoWorld Pointprobe) at nominal resonance frequency at 320 kHz in the same populations as above.

### Scanning electron microscopy imaging

Cell surface morphology was examined using a scanning electron microscope (SEM) (Hitachi TM3000). Before observations, cultures were fixed in 4% PFA at room temperature. All samples were analysed at 15 kV in low vacuum state for non conductive materials. An energy-dispersive spectrometer (EDX) (Oxford Instruments SwitED) was used in conjunction with SEM for elemental analysis of the deposited mineral phase.

### Electrophysiology

BM-hMSC membrane currents were recorded in the whole-cell configuration of the patch-clamp technique, as previously described^21^. Before recording, cells were detached from the substrate, re-suspended in a Standard Solution containing (in mM): 150 NaCl, 5.4 KCl, 2.0 CaCl_2_ 1.0 MgCl_2_, 10 HEPES, 10 glucose (pH 7.4) and used within 6 h.

Na^+^ and K^+^ currents were recorded in the Standard Solution described above. Blockage of K^+^ currents was elicited with a bath solution containing (in mM): 130 Tetraethylammonium chloride (TEA-Cl), 10 BaCl2, 1.0 Mg2Cl, 10 HEPES, 10 glucose (pH to 7.4 with KOH). Ca^2+^ currents were measured in 108 BaCl_2_ and 10 HEPES, (pH 7.4). Patch electrodes had tip resistance 3.0–5.0 MΩ when filled with a solution containing (in mM): 8 NaCl, 40 KCl, 100 Aspartic Acid, 100 KOH, 2 CaCl_2_, 5 EGTA and 4 adenosintriphosphate (ATP). GEPULSE software was used for current acquisition. [http://users.ge.ibf.cnr.it/pusch/programs-mik.htm]

### Confocal microscopy

Cells were fixed with 4% (w/v) PFA for 1 h at 4 °C, washed with PBS three times for 15 min and blocked by incubating with PAT (PBS containing 1% [w/v] BSA and 0.02 [v/v] Tween 20) for 2 h at room temperature^17^,^22^. Anti-type I collagen, anti-decorin, anti-osteopontin, and anti-alkaline phosphatase (ALP) rabbit polyclonal antisera, (provided by Dr. Larry W. Fisher National Institutes of Health, Bethesda, MD), were used as primary antibodies diluted 1:500 in PAT. The incubation with primary antibodies was prolonged overnight at 4 °C and the negative controls were incubated with PAT alone. After washing, samples were incubated with Alexa Fluor 488 goat anti-rabbit IgG (Molecular Probes) at a dilution 1:750 in PAT for 1 h at room temperature. Cells were counterstained for 15 minutes with a solution of Hoechst (2 μg/mL; Sigma-Aldrich) to target nuclei and examined under a confocal laser scanning microscope (CLSM) model TCS SP2 (Leica Microsystems, Bensheim, Germany).

### Epifluorescence imaging

To evaluate L-type Ca^2+^ channel expression, cells were fixed in 4% PFA at room temperature, permeabilized with 0.2% Triton X-100 and blocked with 20% normal goat serum (Vector, Labs Burlingame, CA, USA) for 1 h at room temperature. Then cells were incubated with rabbit anti-human L-type α1C subunit (CaV1.2) (1:100, Santa Cruz Biotechnology)^23^ and mouse anti-human osteocalcin (1:50 Santa Cruz Biotechnology), washed and incubated with Alexa-fluor-488 (green) goat anti-mouse and Alexa-fluor 647 (red) goat anti-rabbit respectively (Molecular Probes). Fluorescence was acquired with a Nikon Di-U upright microscope equipped with a Nikon DS-Qi1 digital CCD camera.

### Intracellular calcium measurements

BM-hMSCs grown on 20 mm diameter coverslips were loaded with Fura-2 AM in the presence of Pluronic F-127 (Sigma-Aldrich GmbH) for 45 min at 37 °C. The time–course of the cytosolic calcium concentration was performed at room temperature in a Standard solution containing (in mM): 150 NaCl; 5 KCl; 2 MgCl_2_; 10 glucose; 10 HEPES; 2 CaCl_2_, pH 7.3.

The basal intracellular free calcium concentration, [Ca^2+^]_i_ was estimated from Fura 2-AM fluorescence using dual-wavelength excitation (340 and 380 nm) and by acquiring a single emission (510 nm). Fluorescence ratio calculation and calibration were performed as previously described^24^.

### Statistics and Data Analysis

Electrophysiological traces were initially analysed by the free software ANA [http://users.ge.ibf.cnr.it/pusch/programs-mik.htm]. All subsequent analysis work and graphing was performed using Sigma Plot (SPSS Science, Chicago IL, USA) software. Data are shown as mean ± standard error (SE).

Boltzmann equation was used to obtain steady-state activation parameters of voltage-gated currents:


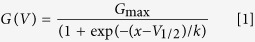


where G_max_ is the maximal conductance, V is membrane potential, V_1/2_ is the potential at which the conductance is half the maximum value and k is a slope factor.

The statistical significance of the differences observed between various experimental groups was calculated using a two-tailed t-test. The p-values of <0.05 were considered to be statistically significant.

The measured intracellular calcium value in every acquisition step was the average of 30 cells present in the field of view of the system, then all data were plotted as the average of 3 acquisitions with standard deviations. Experimental data for each cell population were fitted by a nonlinear regression, according to the following equation:


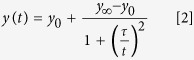


a typical four parameters sigmoidal curve. In our experiments:


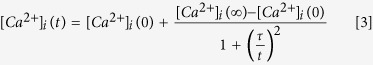


where [Ca^2+^]_i_ (t = 0) is the calcium concentration in undifferentiated cells, [Ca^2+^]_i_ (t = ∞) is the calcium concentration in fully differentiated cells, τ delineates the speed of the differentiation process.

## Results

To investigate the functional and structural consequences of BM-hMSC exposure to a low frequency PEMF during osteogenic differentiation, four different cell populations were monitored: a) control BM-hMSCs grown in Maintenance Medium (MM) up to 27 days without exposure to PEMF (ODM−/PEMF−); b) cells grown in MM up to 27 days and daily exposed to PEMF for 10 minutes (ODM−/PEMF+); c) cells differentiated up to 27 days in Osteogenic Differentiation Medium (ODM) (ODM+/PEMF−); d) cells differentiated up to 27 days in ODM and daily exposed to PEMF (ODM+/PEMF+).

### Cell viability

Average cell viability was evaluated at days 3, 9, 15, 21, and 27 in culture for all the above treatments using MTT. For all conditions, viability was in the range of 88%–95% with no statistically significant differences between samples (p > 0.05) (data not shown).

Annexin V and propidium iodide (PI) staining was performed, on the same days as the MTT test. Confocal laser scanning microscopy (CLSM) analysis showed that all tested cells were negative (data not shown), indicating that PEMF exposure did not induce cell apoptosis.

### Morphological modification of BM-hMSCs in response to osteogenic differentiation and PEMF exposure

**Figure 1:**
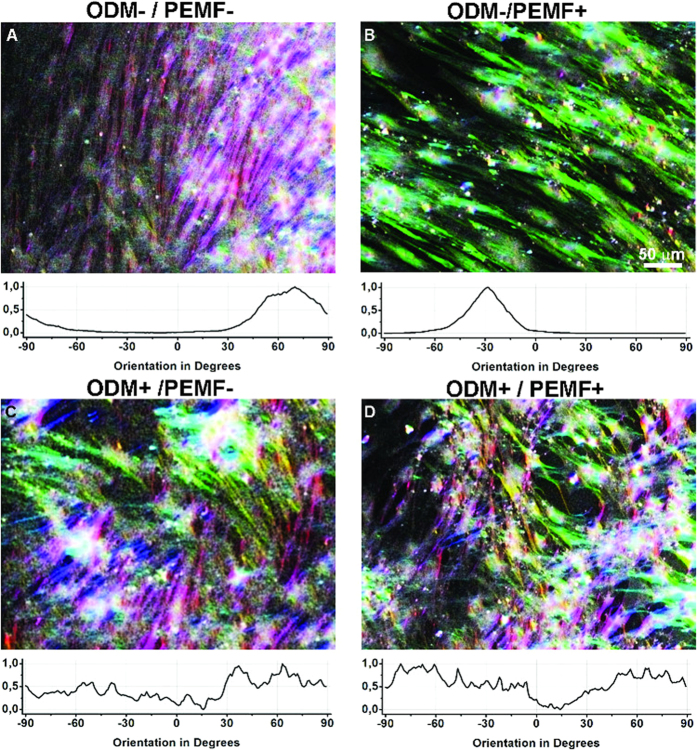
Orientation analysis provides information on morphological modifications in BM-hMSCs. Optical images of cells at confluence highlight a different organization between untreated ODM− (first row **A**,**B**) or ODM+ treated (second row, **C**,**D**) samples exposed (PEMF+) (first column, **B**,**D**) or not (PEMF−)(first column, **A**–**C**) to PEMF. Image colours encode the local orientation, so that univocally oriented cell populations result in almost monochromatic images, while multicolour pictures are associated to spread-out and unordered populations. The graph under each image is a representation of orientation distribution. The presence of a peak (**A**,**B**) indicates the existence of a prevalent direction.

BM-hMSCs grown in sparse culture showed remarkable morphological variability due to the lack of homogeneity in the population, which is composed of a mixture of undifferentiated cells including committed progenitors^25^. This variability decreased when undifferentiated BM-hMSCs reached confluence and started to organize into a more homogeneous culture, showing elongated soma and a uniform preferential local orientation (Fig. 1A). Conversely, when differentiation had progressed further with the addition of chemical inducers, BM-hMSC morphology underwent reorganization, resulting in an osteo-like culture composed of flat cells widely spread on the growing surface, of irregular shape and undefined boundaries (Fig. 1C and^4^). This property, which was easily detectable by visual inspection and robustly reproducible, was quantified by means of orientation analysis. As briefly described in the Materials section, local orientation was calculated for each image and the resulting distribution was plotted in Fig. 1 graphs. Undifferentiated cells (ODM−/PEMF−), which showed a tendency to align, are in fact characterized by a uniform orientation distribution with a clear peak corresponding to the main direction in the field of view (graph of Fig. 1A), while differentiated cells (ODM+/PEMF−) present a broader distribution with no leading orientation peak (graph of Fig. 1C). Interestingly, Fig. 1B,D and related graphs show that, regardless of the original morphology, PEMF exposure does not cause any change in cell orientation, thus in cell shape, during proliferation or differentiation (ODM−/PEMF+, ODM+/PEMF+).

### Deposition of extracellular Calcium

Modifications in the cellular phenotype were followed during differentiation and monitored by means of optical imaging and atomic force microscopy (AFM).

**Figure 2:**
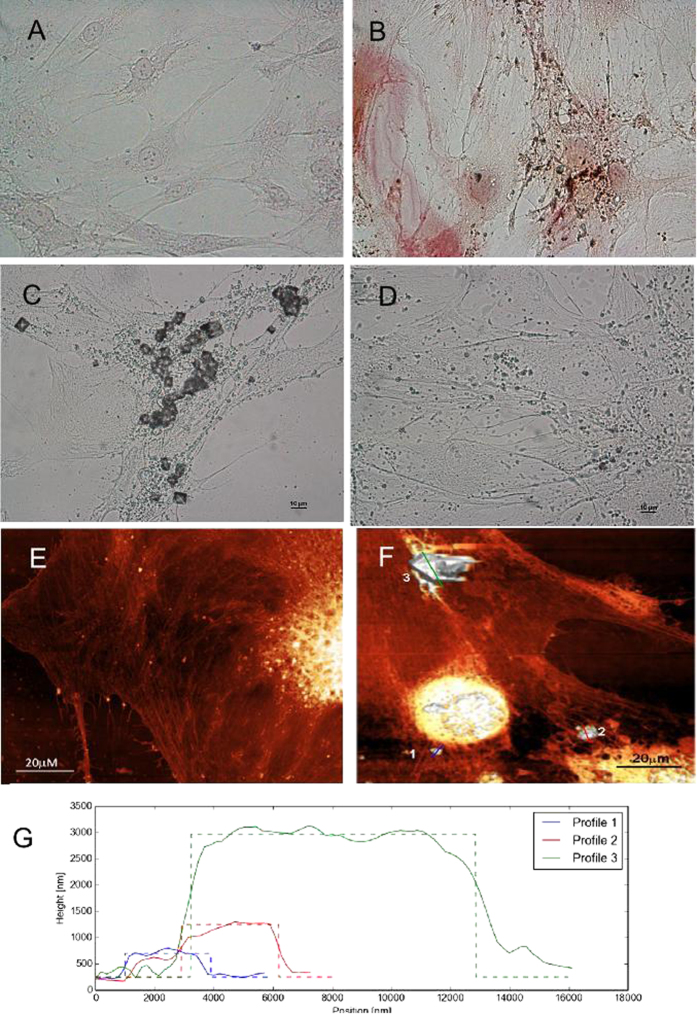
Osteo-differentiation of BM-hMSCs. (**A**,**B**) Alizarin Red staining: (**A**) ODM−/PEMF−; (**B**) ODM+/PEMF− BM-hMSC cells. Red precipitates accumulate in correspondence with calcium produced by the cells. Selected images are representative of three independent experiments. Similar results were also obtained in ODM+/PEMF+. (**C**,**D**) Different quantities of aggregates of various sizes are detectable in the culture medium and on the cell surface after 27 days of ODM+ (**C**) or ODM+/PEMF+ treatment (**D**). (**E**–**G**) AFM analysis: AFM contact mode images of ODM−/PEMF− (**E**) and ODM+/PEMF− (**F**) Special structures are identified by numbers and the corresponding profiles are reported in panel (**G**). The bars are 20 μm long. Images were obtained in air environment.

BM-hMSCs cultured in ODM for 27 days showed Alizarin red staining, commonly associated to the establishment of an osteo-like phenotype (Fig. 2B), while no coloration was detected in the control sample (Fig. 2A). During differentiation, a few deposits started to appear on cell surface and in solution (see Fig. 2C,D). PEMF exposure had a negative effect on the production of Calcium deposits: considerably fewer deposits were observed in ODM+/PEMF+ samples (Fig. 2D) than in ODM+/PEMF− samples (Fig. 2C). Control ODM−/PEMF− cells did not produce any detectable deposit even after 27 days in culture (data not shown).

Detailed analysis performed by AFM imaging (see Fig. 2E–G) confirmed the presence of particles on the surface of differentiated cells (Fig. 2F), making it possible to distinguish between two classes with different morphological characteristics. One class consists of particles showing a porous amorphous surface (structures 1 and 2 in Fig. 2F), with a height of about 1 μm (red and blue profiles Fig. 2G), while the other (structure 3 in Fig. 2F) consists of particles that are over three times higher and exhibit a regular, smooth crystal-like structure (green profile Fig. 2G).

**Figure 3:**
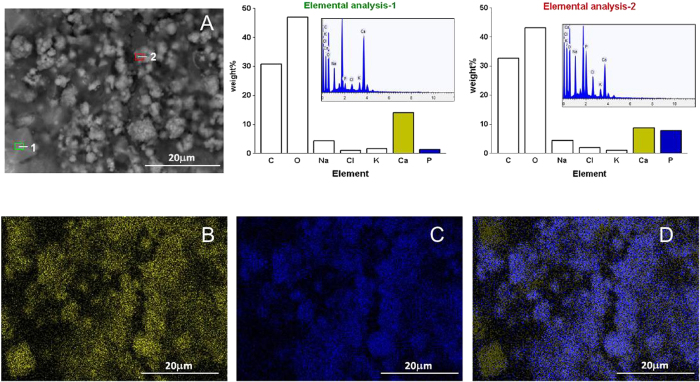
SEM/EDX analysis of Calcium deposits. (**A**) SEM picture showing different types of Ca^2+^ deposits in BM-hMSCs treated for 27 days with ODM (ODM+/PEMF−). Elemental analyses 1 and 2 refer to the regions highlighted in image (**A**), respectively concerning the big crystal-like particle and the smaller amorphous particle. A high peak relative to phosphorus is present in particle 1 (see spectrum 1 in the inset), but absent in particle 2 (see spectrum 2 in the inset). The percentage weights of each element of the spectra are reported in the related histograms. Below: EDX elemental mapping of the distribution of Calcium (**B**), Phosphorus (**C**) and overlapped Calcium-Phosphorus (**D**) relative to the picture in (**A**). The bright yellow areas contain mostly Calcium, while the bright blue areas contain mostly Phosphorus.

To gain a better insight into the chemical nature of the deposits, SEM-guided Energy-dispersive X-ray spectroscopy (EDX) analysis was performed on selected samples after ODM and/or PEMF exposure (Fig. 3). The two types of deposits described in Fig. 2 were clearly identified by SEM imaging, and EDX chemical analysis made it possible to determine their elemental composition. Both structures have a strong calcium signal (Fig. 3A), but the crystal-like particles lack the phosphorus peak (elemental analysis 1). This observation indicates that the bigger crystal-like particles are calcium salts, probably calcium oxalate, while the smaller amorphous aggregates, which contain phosphorus and calcium, correspond to hydroxyapatite (HA)^26^. The chemical analysis was directly visualized by means of EDX elemental mapping (Fig. 3B–D) in which calcium and phosphorus concentrations were mapped in yellow and blue respectively, demonstrating that large crystals such as particle 1 in Fig. 3A do not have the phosphorous signal present in the amorphous, HA-rich structures.

### Electrophysiology: undifferentiated BM-hMSCs

In order to gain substantial information about the ion channel pool expressed in BM-hMSC, patch clamp experiments were performed by recording currents in the whole-cell configuration on single cells between passage 3 and 8.

**Figure 4:**
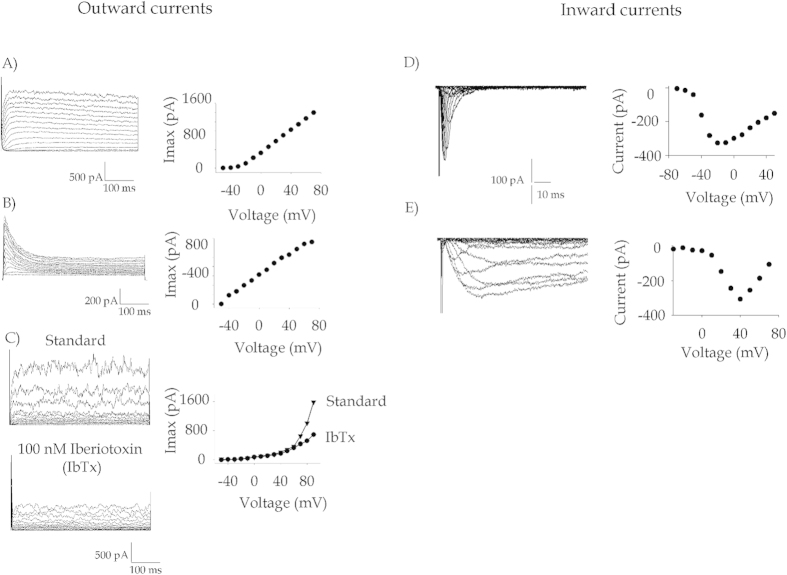
Families of voltage-gated ion channels expressed in control BM-hMSCs. (**A**–**C**) Representative traces of voltage-activated outward currents, identified as K^+^ currents. Channels were stimulated by applying 300 ms long depolarizing steps from −90 to +80 mV in 10 mV increments, starting from a V_hold_ (holding potential) of −90 mV. The corresponding current-voltage relationship (I–V) is reported to the side of each series of traces. (**D**,**E**) Traces of voltage-gated inward currents recorded in control BM-hMSCs. (**D**) Transient inward currents were elicited in standard ionic conditions. Steady-state activation parameters were calculated assuming a reversal potential of +90 mV and fitted to a Boltzmann equation (equation [1]). In this cell V_1/2_ = −30.9 mV and =11.26. Similar results were found for the other cells. (**E**) A slower, more persistent inward current was elicited by applying 100 ms long pulses in Na^+^-free conditions and in the presence of Ba^2+^ only as charge carrier. Boltzmann parameters of Ca^2+^ currents are reported and explained in the text.

In voltage-clamp conditions and in the presence of K^+^ in the pipette, all tested BM-hMSCs showed outward rectifying voltage-activated currents. Multiple current profiles were expressed in the cells, as shown in Fig. 4A–C, which illustrate currents in representative cells activated with the voltage protocol described in the legend. In particular, panel A in Fig. 4 shows a gradually activating current with a threshold around −20 mV and a plateau at highly depolarizing voltages (see I–V relationship on the left). This current, which was expressed by the vast majority of the tested cells, was significantly inhibited by TEA-Cl (data not shown) and was identified as a delayed rectifier K^+^ current^27^.

In Fig. 4B, a fast inactivating component appears in the outward K^+^ current. For this current, the threshold of activation was −40 mV and the decay phase was fitted by a single exponential curve with a time constant of 45 ms.

In Fig. 4C, traces of a noisy oscillating outward current are represented (upper traces) together with the relative current-voltage plot. The oscillations and the current were proved to be associated to Maxi-K^+^ channels, a family of K^+^ channels activated by intracellular calcium^28^, a finding that is corroborated by the observed reduction upon treatment with the specific blocker Iberiotoxin (lower traces). In unstimulated BM-hMSCs, the fraction of cells showing the noisy component was about 65%, the fast inactivating current was expressed only in 24% of cells and almost every cell showed KDR currents. In general, more than one K^+^ conductance coexisted in each individual BM-hMSC, and this mixture further increased the variety of observed current profiles.

Inward currents were first detected by maintaining the cells in the same ionic condition as for K^+^ and by applying 12 ms long voltage pulses from −50 to +50 mV in 10 mV steps. This protocol highlighted a fast inward transient current (Fig. 4D), which activates at around −30 mV, reaches its maximum at +20 mV, and is reversibly inhibited by 2 μM Tetrodotoxin (TTX, data not shown). This inward current, expressed in about 60% of control BM-hMSCs, could be identified as a voltage-activated TTX-sensitive Na^+^ current.

After switching to a stimulation protocol including 100 ms long pulses from −60 to +60 mV (V_h_ = −100 mV), and using 108 mM BaCl_2_ as the only charge carrier, a slowly inactivating inward current was elicited in a fraction of BM-hMSCs (Fig. 4E), clearly recalling the behaviour of the voltage-gated calcium current (VGCC)^10^.

### Electrophysiology: BM-hMSCs exposed to Osteogenic differentiation and PEMF. Effects on VGCC properties and expression

Patch clamp recordings were repeated at selected time points (3, 9, 15, 21, 27 days of treatment) for all conditions (ODM−/PEMF; ODM−/PEMF+; ODM+/PEMF−; ODM+/PEMF+) to gain a complete picture of the BM-hMSC ion channel pool during osteogenesis and PEMF exposure.

**Figure 5:**
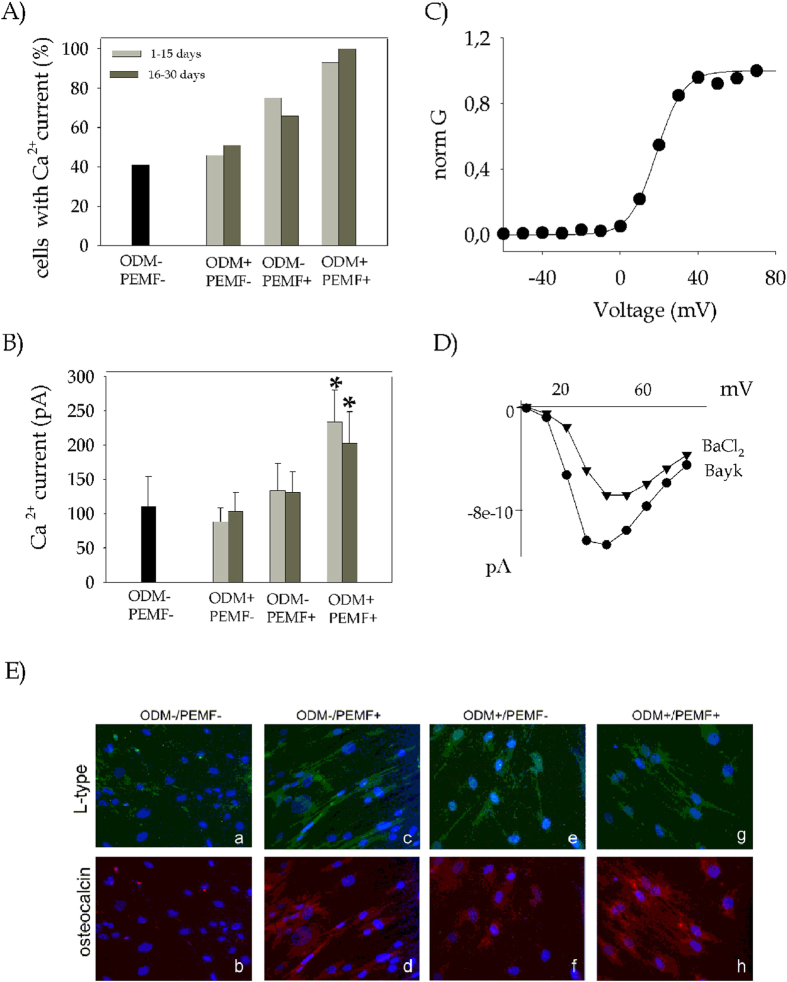
Voltage-gated Ca^2+^ currents are enhanced by ODM treatment and even more markedly by PEMF exposure. Electrophysiology. (**A**) Histogram showing the percentage of cells expressing Ca^2+^ current, in the four different populations tested. (**B**) Histogram representing the mean Ca^2+^ current amplitude. Values are plotted as mean ± SE. The significance of the differences obtained is reported as *(P < 0.05) (**C**) Voltage dependence of steady-state activation of Ca^2+^ current. Normalized conductances were plotted as a function of voltage and fitted to a Boltzmann equation (see equation [1]). In the representative cell shown in (C), best fit parameters were *V*_*1/2*_ = 18.67 mV and *k* = 6.26. (**D**) Pharmacological analysis of Ca^2+^ currents. Currents were elicited by applying the same protocol described in Fig. 4E and then challenged with BayK8644. Steady-state current amplitude values were reported in the figure as a function of the voltage for the control current and after the addition of BayK. (**E**) Ca^2+^ channel immunodetection. Fluorescence microscopy images showing localization of L-type VGCC (green) and Osteocalcin (red). Nuclear chromatin was stained with DAPI (blue). The bars are 100 μM in length.

The behaviour of K^+^ and Na^+^ currents described in the previous section did not change significantly in any of the conditions throughout the 27 days of culture, while the Ca^2+^ currents exhibited several differences despite the overall similarities. Firstly, the presence of cells expressing Ca^2+^ currents increased significantly during differentiation. As shown in in Fig. 5A, no more than 40% of the unstimulated BM-hMSC controls (ODM−/PEMF−) expressed detectable Ca^2+^ currents, whereas ODM+/PEMF− cells showed a slight increase, which became substantial for ODM−/PEMF+ and peaked in ODM+/PEMF+. At the same time, the average amplitude of Ca^2+^ currents per cell significantly increased in ODM+/PEMF+ conditions (lower histogram, 5B). This effect, independent of membrane capacitance, after the first 9 days of treatment produced an average current of 199 ± 68 pA, n = 8, almost double the current elicited in the other treatments (70 ± 13, n = 13, for ODM+/PEMF−; 113 ± 45, n = 4, for ODM−/PEMF+). This was still observed at day 15 and at day 27, when the average Ca^2+^ current was 245 ± 7 pA (n = 9) in ODM+/PEMF+, 125 ± 29 (n = 20) in ODM−/PEMF+, 106 ± 29 (n = 13) in ODM+/PEMF-.

In order to assess the molecular identity of the ion channels responsible for Ca^2+^ currents in BM-hMSCs, their biophysical properties were further investigated during the differentiation process. Current traces were recorded by applying the protocol for Ca^2+^ currents described in Fig. 4E using Ba^2+^ as charge carrier; steady-state activation parameters were estimated by plotting the peak values of Ca^2+^ conductance versus voltage, and fitting them with the Boltzmann equation (equation [1]) (Fig. 5C). Interestingly, neither the activation voltage (V_1/2_) nor the slope factor (k) were significantly different even after 27 days of ODM+/PEMF+ treatment as compared to the control sample. Averaged V_1/2_ and k were 19.09 ± 2.09 mV and 6.89 ± 0.81 for ODM−/PEMF− sample (n = 7) and V_1/2_ = 20.49 ± 0.70 mV and k = 7.25 ± 0.34 for ODM+/PEMF+ (n = 8), thus suggesting that osteogenesis only affects channel expression level and not channel type. Similar results were also obtained for the ODM+/PEMF− and ODM−/PEMF+ samples (data not shown). Challenging Ba^2+^ currents with 10 μM Bay-K8644, a modulator of L-type VGCC^10^, caused a current potentiation of approximately 170% (n = 31), in agreement with previous results^29^. The results shown in Fig. 5A,B point at L-type VGCC as putative targets of PEMF activity during hMSC osteogenesis; indeed, PEMF exposure (see the ODM−/PEMF+ sample) seems to drive VGCC transcription and assembly more intensely than the addition of hormones and chemical agents to the culture medium (see ODM+/PEMF−). Electrophysiology results were further supported by immunocytochemistry, by targeting the α1C subunit of L-type VGCC (green stained, Fig. 5E)^30^, togheter with osteocalcin (red stained), a marker expressed during the mineralization phase in rat calvarial osteoblasts^2^. Control BM-hMSCs showed no red fluorescence and a few cells showed green fluorescence (Fig. 5E, a,b), while both levels increased in the presence of PEMF and/or ODM (Fig. 5E c–h), confirming the electrophysiological observations.

### Immunodetection of osteogenic markers

**Figure 6:**
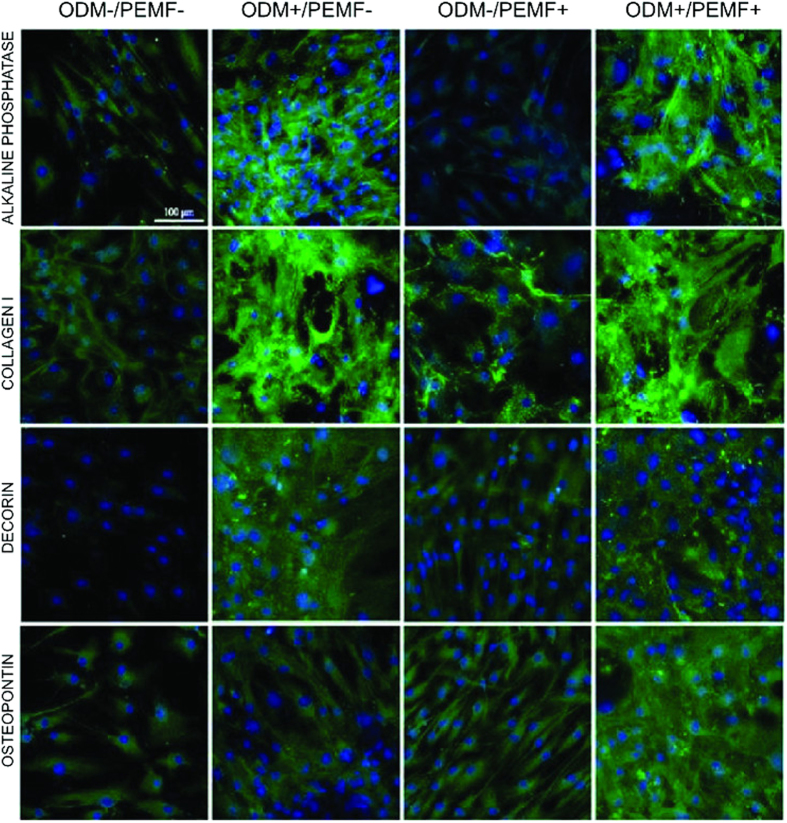
Immunodetection of osteogenic differentiation markers: effect of ODM treatment and PEMF exposure for BM-hMSCs grown up to 27 days. Localization of specific osteogenic markers, alkaline phosphatase, collagen type I, decorin and osteopontin, performed on the same samples as in Fig. 5E. Staining confirmed that the osteogenic phenotype is triggered by ODM treatment (columns 2 and 4) and is maintained by PEMF exposure (column 4) or even slightly incremented (see osteopontin).

Immunolocalization tests were employed in order to highlight the expression of typical putative osteogenic proteins such as alkaline phosphatase, collagen type I, decorin and osteopontin in all experimental conditions (see Fig. 6). At the end of the culture (day 27), ODM treatment provided the expected onset of an osteo-like phenotype (see ODM+/PEMF− column in Fig. 6), and its activity seemed to be maintained or even slightly potentiated (see osteopontin, ODM+/PEMF+ column) when applied in combination with PEMF.

### Intracellular calcium measurements

**Figure 7:**
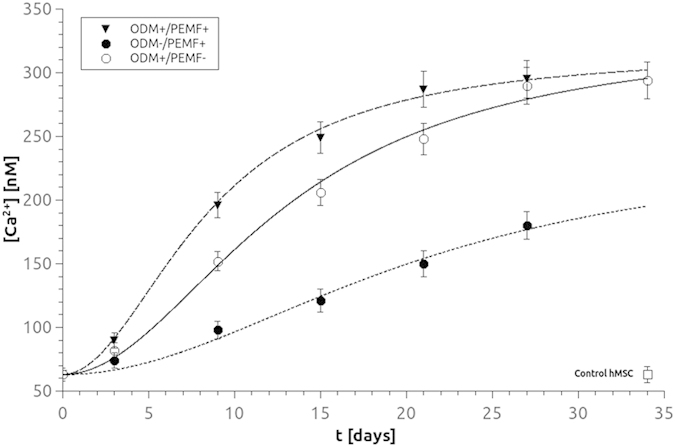
Estimation of intracellular level of [Ca^2+^]_i_: effects of ODM treatment and PEMF exposure for BM-hMSCs grown up to 34 days. Mean time-response curves obtained from ODM+ /PEMF+ (▼), ODM−/PEMF+ (●), or ODM+/PEMF− (○) samples indicate a synergistic effect deriving from the simultaneous application of the two treatments. As a negative reference, the value of BM-hMSC cells grown 34 days in MM (ODM−/PEMF− (⬜) is shown. Each point represents the mean of 3–6 distinct experiments. Data fits have been performed according to equation [3] (see text, Material and Methods section).

A quantitative assay for the measurement of intracellular basal calcium concentration [Ca^2+^]_i_ was employed to obtain a direct measure of the action of specific effectors on osteodifferentiation (Fig. 7).

The basal calcium concentration in BM-hMSCs under each different condition was measured at selected time points (3, 9, 15, 21, 27 and 34 days, Fig. 7). All data were plotted as the average with standard deviations (ave ± SD, n = 30). Noticeably, [Ca^2+^]_i_ values for ODM+/PEMF− treatment (empty circles in Fig. 7) show a monotone increase as a function of time, suggesting a correlation between basal intracellular calcium value and differentiation stage. Experimental data were fitted by a nonlinear regression, according to equations 2 and 3 reported in the Materials and Methods section.

Experimental results for ODM+/PEMF− were fitted by the parameter values [Ca^2+^]_i_ (t = 0) = 63 ± 3 nM, [Ca^2+^]_i_ (t = ∞) = 330 ± 10 nM, τ = 13 ± 2 days. The value of basal calcium at t = 0, [Ca^2+^]_i_ (t = 0) = 63 ± 3 nM, obtained for ODM+/PEMF− was then set as a parameter for all fits in subsequent conditions.

The basal value of [Ca^2+^]_i_ was measured in ODM−/PEMF+ BM-hMSC (full circles). A very slow but constant increase in [Ca^2+^]_i_ was still observed, indicating Ca ^2+^ signaling as a possible target of magnetic field action, in agreement with previous data^31^; nevertheless the final value was remarkably lower than in previous conditions. Results were fitted with [Ca^2+^]_i_ (t = ∞) = 247 ± 9 nM and τ = 21 ± 3s. Finally the basal value of [Ca^2+^]_i_ was measured for BM-hMSCs in ODM+/PEMF+ conditions (filled triangles), showing the fastest increase. Data were fitted with τ = 8 ± 2 and [Ca^2+^]_i_ (t = ∞) = 317 ± 11 nM.

As a reference, we verified that in BM-hMSCs grown for the same period of time in the absence of any stimulation (ODM−/PEMF−), [Ca^2+^ ]_i_ did not undergo any modification (63 ± 5nM, empty square).

## Discussion

The general aim of this work was to address the effects of *in vitro* PEMF exposure on osteodifferentiation of mesenchymal stem cells isolated from human bone marrow (BM-hMSC). Several biochemical tests reported in the literature were applied in order to verify the differentiation stage, even though none of them proved to be fully osteo-specific^32^,^33^. To obtain an unambiguous, quantitative characterization of cell populations, a panel of biophysical tools were adopted and tailored for BM-hMSC differentiation.

A comparative analysis of cell morphology was performed, revealing a significant level of heterogeneity. Control unstimulated BM-hMSCs are mostly fibroblast-like and spindle-shaped, and remain unchanged when samples are exposed to PEMF only over a period of 27 days. Conversely, cells grown in the ODM are polygonal and spread, even after PEMF stimulation. This change in *“in vitro”* morphology might be related to the addition of dexamethasone to the culture medium^34^, but undoubtedly the resulting cell shape closely resembles osteoblasts^4^, representing in itself a good indicator of differentiation. Local alignment of BM-hMSC populations was chosen as a feature strictly related to cell shape and was quantified by means of an orientation analysis protocol. The frequency histograms, utilized to indicate whether or not a cell population had an orientation, clearly distinguished between ODM+ and ODM− populations and, most importantly, showed that PEMF does not affect cell shape regardless of the original morphology of the exposed cells.

According to the model proposed by Owen and collaborators [1990], mineralization of the extracellular matrix starts near the midpoint of osteogenic differentiation in rat calvarial cells. After several days of ODM exposure, BM-hMSCs produce and release aggregates containing Calcium and Phosphorus, recognized by EDX analysis as hydroxyapatite. Aggregates are abundant in ODM+/PEMF− cells, and decrease in ODM+/PEMF+, thus corroborating the hypothesis that PEMF is more effective during the differentiation stage and on specific pathways only, whereas it only plays an inhibitory role during the mineralization phase^35^. The presence of HA, usually organized as a lattice in the extracellular matrix of mature bone, is an unequivocal indication of BM-hMSC osteodifferentiation.

The changes in [Ca^2+^]_i_ and in the array of ion channels expressed were also monitored during BM-hMSC differentiation and showed the gradual development of a calcium metabolism more similar to osteoblasts than to the progenitor cells.

For untreated BM-hMSC cells, our results confirm the expression of Na^+^, Ca^2+^ and K^+^ voltage-activated currents that are important to stem cell proliferation, migration^36^, growth and mineralization^28^, but also reveal that none of them seems to undergo significant modifications during osteogenesis. Conversely, considerable differences are observed in L-type VGCC expression, which mediates Ca^2+^ influx in response to membrane depolarization. Mean peak current amplitude increases following stimulation, and the percentage of cells encoding functional L-type VGCC shifts from 40% of untreated BM-hMSC cells to 100% in ODM+/PEMF+ cells treated for 27 days.

A gradual, persistent rise in [Ca^2+^]_i_ concentration, from 63 to 300 nM, is observed. In agreement with the trend observed for extracellular Ca^2+^ deposition, PEMF seems to influence [Ca^2+^]_i_ in the early period of differentiation mainly: after 9 days of exposure, [Ca^2+^]_i_ is 30% higher in ODM+/ PEMF+ than in ODM+/ PEMF− cells and later it brings forward its plateau value by about 7 days as well (Fig. 7). It is known from the literature that the basal level of intracellular calcium in osteoblasts is in the range of 50–150 nM^37^ and that when the cell is stimulated by a pharmacological agent this value rises up to 400 nM or above. The values estimated for our BM-hMSCs in differentiation indicate a persistent elevation of [Ca^2+^]_i_ which does not fit into the above model. However, one possible explanation may be that, in order to maintain the plasticity required to mediate long-term changes associated with differentiation, these cells require the presence of a high level of free [Ca^2+^]_i_ for downstream Ca^2+^-pathways^38^.

The different trends observed between [Ca^2+^]_i_ variation (Fig. 7) and VGCC expression (Fig. 5) throughout hMSC osteogenesis may appear inconsistent and thus deserve further comment. A conspicuous number of papers support the idea that L-type VGCCs are the main targets of PEMF action (see refs 16, 31), whereas others partially disagree (39), though recognizing a lesser role of VCGGs in the cellular response to PEMF (39). Accordingly, our results demonstrate that PEMF exposure during hMSC osteogenesis has a stronger impact on expression of functional VGCCs than the addition of chemical agents (ODM+) to the culture medium (see Fig. 5). Conversely, PEMF exposure does not bring about the same level of [Ca^2+^]_i_ increase as the addition of ODM does (see Fig. 7). As already postulated by Kawano and collaborators^39^, this finding is not unexpected since VGCCs cannot be the only reason for the increment in [Ca^2+^]_i_ which represents the combined effect of all Ca^2+^-related pathways in BM-hMSCs.

Even though different pathways correlate with [Ca^2+^]_i_ modification during differentiations, L-type VGCC are abundantly expressed in osteoblasts, where they have a role in gap junction-dependent propagation of Ca^2+^ signalling^40^. Thus further experiments on intracellular calcium stores and Ca-permeable ion channels other than L-type VCGG are necessary in order to identify all the actors that play a role in Ca^2+^ homeostasis while hMSC osteogenesis is under way.

## Conclusions

The results obtained confirm that PEMF affects BM-hMSC osteo-differentiation by favouring the early stages of osteogenesis and is selective for specific pathways. At least two molecular mechanisms seem to be stimulated by PEMF application during osteogenesis: expression of L-type VGCC and modulation of the concentration of cytosolic free Ca^2+^. Both pathways are positively affected by PEMF exposure as well as by treatment with ODM; the two stimulations operate synergically when applied simultaneously and are likely to act through different mechanisms in multiple and sometimes distinct metabolic pathways. Thus the combined treatment could be regarded as an adequate protocol for *in vitro* studies of early stages of osteogenesis.

## Additional Information

**How to cite this article**: Petecchia, L. *et al.* Electro-magnetic field promotes osteogenic differentiation of BM-hMSCs through a selective action on Ca^2+^-related mechanisms. *Sci. Rep.* **5**, 13856; doi: 10.1038/srep13856 (2015).
